# Glial regenerative cell types in the superficial cortex in cortical dysplasia subtypes

**DOI:** 10.1016/j.eplepsyres.2020.106529

**Published:** 2021-01

**Authors:** Joan Y.W. Liu, Cheryl Reeves, Rianne van der Pijl, Maria Thom

**Affiliations:** a Department of Clinical and Experimental Epilepsy, UCL Institute of Neurology and National Hospital for Neurology and Neurosurgery, Queen Square, London, WC1N 3BG, UK; b School of Life Sciences, University of Westminster, London, W1W 6UW, UK; c Division of Neuropathology, National Hospital for Neurology and Neurosurgery, Queen Square, London, WC1N 3BG, UK

**Keywords:** FCD, focal cortical dysplasia, HS, hippocampal sclerosis, PDGFRβ, platelet derived growth factor receptor beta, PAX6, paired-box transcription factor 6, Cortical layer I II, Glial regeneration, Cortical dysplasia

## Abstract

**Purpose:**

Focal Cortical Dysplasias (FCD) are localized malformative brain lesions in epilepsy. FCD3a associated with hippocampal sclerosis, affects the superficial cortex and is presumed to have an ‘acquired’ rather than developmental origin. Precursor cells may arise outside neurogenic zones including cortical layer I. Our aim was to characterise subsets of glial progenitor cells in the superficial cortical layers, known to be involved in gliosis and gliogenesis and that could distinguish FCD3a from other subtypes.

**Methods:**

Using immunohistochemistry we quantified the density of glial progenitor subsets in superficial cortex layers using markers against PAX6, GFAP, Olig2 and PDGFRβ and proliferation marker MCM2 in ten FCD3a cases compared to 18 other FCD types and 11 non-FCD controls.

**Key findings:**

Glial progenitor cells types were present in the cortical layer I and II in all FCD groups. GFAP cells frequently expressed PAX6 and significantly higher GFAP/PAX6 than GFAP/MCM2 cell densities were identified in the FCD3a group (p < 0.05). Olig2 cell densities were significantly higher in FCD3b than FCD3a (p = 0.002) and significantly higher GFAP/MCM2 compared to PDGFRβ/MCM2 cell densities were identified in both FCD3b and FCD2 groups. There was no correlation between cell densities and the age of patients at surgery and between cortical regions.

**Significance:**

Immature and proliferative glial populations across FCD variants reflect reactive cell types and differences may provide insight into underlying pathomechanisms. Higher PAX6 expression in astroglial cells in FCD3a may indicate a switch to astrocytic maturation and enhanced superficial gliosis. Higher Olig2 and GFAP/MCM2 densities in FCD3b may reflect margins of the tumour infiltration zone rather than true cortical dysplasia.

## Highlights

• Cytoarchitectural alterations and gliosis in the superficial cortex characterise FCD type 3a.
• A range of glial-regenerative cell types are represented in layer I/II across FCD types.
• Higher proportions of GFAP/PAX6 than GFAP/MCM2 glial cells characterised FCD3a reflecting switch to glial maturation.

## 1 Introduction

Focal cortical dysplasia (FCD) type 3a is a cytoarchitectural change encountered in approximately 10 % of patients with Temporal Lobe Epilepsy and Hippocampal Sclerosis (TLE/HS). FCD3a principally involves the superficial cortical layers with abnormal clustering of neurones in layer II, reduction of neurones in the lower part of layer II/III and accompanying laminar gliosis (Blumcke et al., 2011). This FCD pattern has been argued to represent an acquired post-natal reorganizational dysplasia, impairing normal maturation, likely to be both aetiologically and temporally linked with the development of HS, although its functional significance remains unclear (Thom et al., 2009). The superficial cortical layers are highly relevant to seizure propagation through extensive cortical-cortical networks and generation of slow rhythms (Halgren et al., 2018) ; discharges from the superficial cortex spread over a larger area than deeper cortex (Serafini et al., 2015), recruitment of layer II/III neurones plays a critical role in seizure spread (Brodovskaya and Kapur, 2019) and activation of cAMP-response element binding protein, a neuronal activity biomarker, was recently shown only in layer II neurones of epilepsy surgical resections (De Santis et al., 2020). There is also a body of evidence of the integral role of astroglia and glial-neuronal interactions in the pathophysiology of epilepsy (Patel et al., 2019) which are less explored in the superficial cortex.

The superficial cortical layers are the last to form during development with complete maturation extending into post-natal period. Experimental studies show deficient Robo1-mediated signalling, that regulates normal neuronal migration to the superficial layers, results in abnormal distribution of neurones in layer II and III during the post-natal stages (Gonda et al., 2013), reminiscent of patterns in FCD3a. Population of reelin-secreting Cajal Retzius neurones persist in layer I into adulthood regulating dendritic complexity (Meyer and Gonzalez-Gomez, 2018) and persistent immature doublecortin-positive neurones are recognised at the interface of layer I and II (Bonfanti and Nacher, 2012) including in TLE/HS, although their physiological function is unclear (Liu et al., 2018a). In addition to immature neurones, glial progenitor cell types are recognised in the superficial cortex and indeed the marginal zone is regarded as a less explored progenitor ‘niche’ during development (Costa et al., 2007). Previously, we have reported populations of developmentally regulated GFAP∂-positive glia in FCD (Martinian et al., 2009) and proliferative nestin-expressing glial cells localised in cortical layer I in TLE/HS (Liu et al., 2018b).

We hypothesised that glial progenitor cell types are integral to both the development and phenotype of FCD3a. Our aim was to evaluate this using paired-box transcription factor (PAX6), which has developmental regulatory roles in differentiation and proliferation of astrocytes (Sakurai and Osumi, 2008), Olig2 transcription factor, regulating oligodendroglial progenitor cells (Mitew et al., 2014) and platelet derived growth factor receptor (PDGFRβ) as a marker for NG-2 progenitor subsets (Garbelli et al., 2015) in FCD3a compared to other dysplasia types and non-FCD controls.

## 2 Methods

39 surgical cases were selected from the archives of the Epilepsy Society Brain and Tissue Bank which included ten representative cases of FCD3a, eighteen cases with other FCD subtypes (FCD1, 2, 3b, 3b, 3d), seven cases with ILAE type I HS and no dysplasia and four non-epilepsy controls ; FCD subtypes were determined using current ILAE criteria (Blumcke et al., 2011) (Table 1). The majority of the resections were temporal cortex and there was consent for use of tissue and ethical approval for this study.

**Table 1:** Table of the 39 cases and quantitative data of layer I/II single labelled cells.

GROUP*	Age at surgery Mean (range) years	Age at seizure onset mean (range) years	Percentage of cases with Febrile Seizures (%)	Associated pathology (Number of cases)	Location	Male (%)	GFAP cell counts / mm^2^	PDGFRβ cell counts / mm^2^	Olig2 cells/ mm^2^	% GFAP MCM2+	% GFAP PAX6+	% PDGFRβ MCM2+
							Mean (SD)	Mean (SD)	Mean (SD)	Mean (SD)	Mean (SD)	Mean (SD)
										[Densities DL cells mean, SD / mm^2^]	[Densities DL cells mean, SD / mm^2^]	[Densities DL cells mean, SD / mm^2^]
**FCD3A**	32 .2	9.3	70 %	HS (10)	T 100 %	50 %	71.41	11.89	**50.4 ^#^**	11.6 %	55%	33 %
**N=10**	(19−47)	(2−17)					(18.76)	(9.8)	**(23)**	(9)	(35)	(11)
										[10,11]	[97.1, 99.0]	[7.6,8.5]
**FCD3B**	28.5	7.9	16 %	DNT/ GG (6)	T 83.3 %	16.7 %	146.1	11.75	**141.3 ^#^**	35 %	73 %	32 %
**N=6**	(22−36)	(1−17)			O 16.7 %		(18.76)	(3.5)	**(100)**	(26)	(19)	(15)
										[56,52]	[62.7,41.6]	[6.8,6.5]
**FCD3D**	24	8	25 %	Neonatal infarct (3), Rasmussen’s Encephalitis (1)	T 100 %	50 %	79.8	4.08	51.98 (24.3)	19 %	53 %	41 %
**N=4**	(17−33)	(3−14)					(23.35)	(5.3)		(11)	(37)	(52)
										[18.7,27.1]	[69.6,61.6]	[2.2,3.4]
**FCD2A/B**	32	6	0%	–	T 60 %	20 %	116.86	12.40	65.2	18 %	31 %	16 %
**N=5**	(18−52)	(3−8)			F 40 %		(63.3)	(8.3)	(52.7)	(14)	(23)	(19)
										[24.3,23.9]	[37.9,40.6]	[2.6, 2.56]
**FCD1A**	5.6	(3−11)	N/A	–	T 77 %	N/A	86.11	**24.76∼**	54.37	4%	82 %	19 %
**N=3**		N/A			O 33 %		(47.9)	**(7.0)**	(8.8)	(2)	(22)	(16)
										[2.87, 2.8]	[62.5,22.3]	[7.5,7.8]
**HS-NO FCD**	42.7	10.2	20 %	–	T 100 %	60 %	138.5	5.83	52.43	9.8 %	61 %	42 %
										(9.6)	(20)	(5)
**N=7**	(35−52)	(4−21)					(83.2)	(4.2)	(34)	{5.8,6.2]	[48.5,24.0]	[4.5,4.1]
**NEC**	39.7	N/A	N/A	Normal cortex for raised ICP (1), adjacent to tumour (3)	T 100 %	100 %	112.9	1.16	N/A	24 %	57 %	33 %
**N=4**	(18−74)						(51.0)	(1.3)		(19)	(37)	(57)
										[21.9,28.2]	[54.6, 31.8]	[0.3,0.6]
Sig.			P = 0.05					**p = 0.01∼**	**p = 0.002 ^#^**			

Significance (Sig.) between groups shown in bold (∼Kruskal Wallis test for all groups, # Mann-Whitney test between 2 groups). DL=double labelled, DNT = Dysembryoplastic neuroepithelial tumour, F = Frontal cortex, FS = febrile seizures, FCD = Focal cortical dysplasia, GG = Ganglioglioma, ICP = raised intracranial pressure, HS = hippocampal sclerosis, N = non epilepsy controls, N/A = data not available or not applicable, T = temporal lobe, O = occipital cortex, SD = Standard deviation. *not all cases were analysed for all immunostains and olig2 IHC was not carried out in NEC group.

**Fig. 1:**
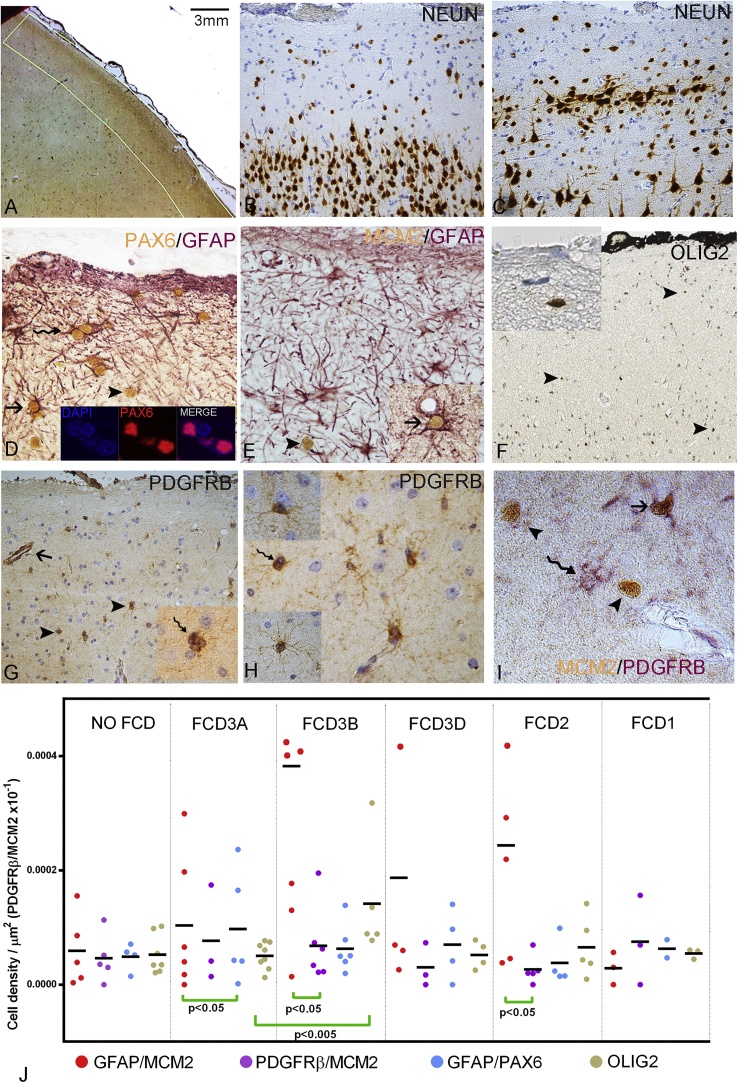
Glial progenitor cell types in the superficial cortical layers of FCD cases. A. Superficial cortex showing the region of interest drawn to include cortical layer I and II of each case; this is an epilepsy control case with no dysplasia, so the middle temporal gyrus, inked in black along the meninges, was examined. B. NeuN labelling in a case with no dysplasia in the superficial cortex compared to (C) a case with the typical features of FCD3a (or temporal lobe sclerosis) with clustering of neurones in layer II and gaps in the lower part of the layer. D. In FCD3a, PAX6 showed nuclear labelling (inset shows co-labelling of PAX6 with nuclear counterstain, DAPI) in a high proportion of GFAP-labelled cells in the superficial cortex (arrow), including multinucleated cells (wavy arrow). Occasionally, PAX6-labelled cells were GFAP-negative (arrowhead). E. In FCD3a, co-labelled MCM2/GFAP (arrow; inset) and single labelled MCM2 cells (arrowhead) were observed. F. Olig2 immunohistochemistry identified small, round cells scattered throughout the superficial layers (arrowheads). G Single (arrowheads) and multinucleated PDGFRβ-labelled cells (wavy arrow in inset) were found in the superficial cortex. PDGFRβ-labelled cells resembling perivascular pericytes (arrow) were not included in the analysis; at higher magnification (H) delicate multipolar processes were apparent on some PDGFRβ-labelled cells (lower inset), and their frequent perineuronal positioning was in keeping with NG2 cell types (top inset) and binucleated cells (wavy arrow). I. PDGFRβ/MCM2 immunohistochemistry revealed single-labelled populations of MCM2 (arrowheads) and PDGFRβ cells (wavy arrow) as well as co-labelled cells (arrow). J. Scatter graph comparing the densities of double-labelled and Olig2 glial populations between FCD and control groups. Bar in A =3 mm and in B, C, F, G = ∼50 microns, E, H = 25 microns, D (and inset) and I = 10 microns.

Immunohistochemistry was carried out on 5 μm formalin-fixed paraffin-embedded brain sections using anti-NeuN (1:100, Millipore, UK), anti-MCM2 (Mini-chromosome maintenance protein 2 for cells licensed for replication, 1:900, BD biosciences, UK), anti-PAX6 (1:100, Santa Cruz Biotech. UK), anti-GFAP (1:1500, DAKO, UK) and anti–PDGFRβ (1:1000, Abcam, UK) as described in previous studies (Liu et al., 2018a; Liu et al., 2018b). Chromogenic double immunolabelling for GFAP/MCM2, GFAP/PAX6 and PDGFRβ/MCM2 was performed using NovaRED Substrate Kit (Vector Lab; Peterborough, UK) and DAKO REAL Envision HRP/DAB kit (Agilent Technologies; Cheshire, UK). The region of interest for analysis was defined on NeuN-labelled sections using an Zeiss Axioscope microscope at x2.5 and the image analysis programme, Histometrix 6 (Kinetic Imaging Limited; Liverpool, UK) to include all of layer I and the upper part of layer II (approximate depth of 3 mm from the pia) of a single gyrus where dysplasia was present. For cases without dysplasia, a similar region of interest along the middle temporal gyrus was examined in each case (Fig. 1A). Manual cell counts of single and double-labelled cells per area (cell numbers/μm2) were performed at 40x magnification for the entire ROI. Quantification was repeated in half of the cases, and the interclass correlation coefficients ranged between 0.92 and 0.95. Perivascular PDGFRβ cells, which may be pericytes, were not included in the counts. Results were compared between FCD subtypes and controls using corrected non-parametric statistical methods (SPSS v25, IBM, New York, USA).

## 3 Results

Qualitative assessment of immunolabelling with NeuN labelling confirmed the subtype of FCD including the typical clustering of neurones in layer II that defines FCD3a as distinct from other FCDs and normal cortex (Fig. 1B-C). PAX6, MCM2, GFAP, Olig2 and PDGFRβ-labelled cells were observed in the superficial cortex in FCD3a (Fig. 1) and also other FCD types (supplemental Fig. 1). PAX6 immunolabelling was exclusively observed within nuclei, as confirmed by immunofluorescent studies and confocal microscopy where PAX6 positive labelling was stained with DAPI, a nuclear marker (Fig. 1D, inset). PAX6/GFAP co-labelled cells, including binucleated cells, were often observed in layer I (Fig. 1D) and GFAP processes were prominent in the immediate subpial layer. MCM2-labelled nuclei were also found to scatter throughout the superficial cortex, sometimes co-labelled with GFAP (Fig. 1E). Olig2 marker also showed nuclear labelling of small cells in layer I and II (Fig. 1F). Single PDGFRβ-labelled small cells were scattered in the superficial layers, many with multiple short processes extending in the parenchyma, some in proximity to neurones and PDGFRβ multinucleated cells were also noted (Fig. 1G,H and insets). PDGFRβ, in addition, labelled pericyte-like cells in close proximity to small vessels, but no labelling of cell processes in the subpial region was observed as with GFAP. A proportion of PDGFRβ-labelled cells were MCM2 positive (Fig. 1).

Quantitative evaluation showed variability in labelling between cases (Table 1). For single labelled cells, significantly higher PDGFRβ cell densities were present in FCD1a than other FCD groups (Table 1; p = 0.01 Kruskal Wallis test) and significantly higher Olig2 cell densities in FCD3b than FCD3a (Table 1; p = 0.002 Mann-Whitney test) (Fig. 1J). For double labelled cells, no significant differences were noted between groups for either cell densities or proportions of double labelled cells (Table 1) although lowest densities were observed in HS cases without dysplasia (Fig. 1J). Wilcoxon rank test for within group differences showed significantly higher GFAP/PAX6 than GFAP/MCM2 densities in FCD3a (p < 0.05); significantly higher GFAP/MCM2 than PDGFRβ/MCM2 densities were present in both FCD3b and FCD2 groups (Fig. 1J). There was no correlation between any cell densities and the age of patients at surgery, age of onset of epilepsy, history of febrile convulsions and no significant differences noted between temporal and extra-temporal regions.

## 4 Discussion

We observed a range of glial progenitor cell types in cortical layer I and II common to FCD types but with differences that could reflect their underlying aetiology. Our aim was to compare FCD3a which primarily involves the superficial cortex to other ‘pan-cortical’ FCD and one finding was higher PAX6/GFAP than GFAP/MCM2 co-labelled cells in FCD3a. PAX6 is a transcription factor and stem cell marker, primarily expressed in radial glia during development with roles in cortical patterning (Ypsilanti and Rubenstein, 2016). In the adult murine cortex, PAX6 is expressed primarily in astrocytes and shown to have a negative effect on cell proliferation, promoting maturation (Sakurai and Osumi, 2008). PAX6 or MCM2 expressing GFAP cells may represent either ‘reactivated mature astrocytes’ or progenitor cell types and increased PAX6-expressing astroglial cell densities in the vicinity of acute cortical injuries were detected in our previous studies (Goc et al. 2014) suggesting they represent regenerative and dynamic cell populations. The frequent bi-nucleation of PAX6-expressing cells in layer I of FCD3a cases may also reflect recent cell division, similar to observations with GFAP∂ labelling (Martinian et al., 2009). The predominance of GFAP/PAX6 over GFAP/MCM2 in FCD3a may reflects a shift towards glial maturation, reflected in the marked superficial cortical gliosis associated with neuronal re-organisation in this pathology (Thom et al., 2009).

PDGFRβ is known to be expressed in brain parenchymal cells other than pericytes, including NG-2 cells, and show dynamic changes in number, proliferation and distribution following seizures and cortical injury (Garbelli et al., 2015; Kyyriainen et al., 2017; Reeves et al., 2019). In the current study, higher PDGFRβ cell densities in FCD1a were noted, but as the numbers within this subgroup was small, the significance of this requires further investigation, particularly since the proportion of proliferative (MCM2 positive) PDGFRβ cells was overall low in this group (Table 1). The proliferative fractions of PDGFRβ cells were higher in FCD3a than FCD2 but at similar levels to TLE/HS without dysplasia. Significantly lower densities of PDGFRβ/MCM2 compared to GFAP/MCM2 cells were noted in both FCD2 and FCD 3b; although PDGFRβ parenchymal cells have been reported in FCD2 the lower proliferative fractions could suggest a less significant role of these progenitor cells in this dysplasia type (Garbelli et al., 2015). It is recognised that low-grade epilepsy-associated tumours can widely extend along the subpial layer and layer I in the adjacent cortex (Blumcke et al., 2019); the significantly higher Olig2 and GFAP/MCM2 densities noted in FCD3b could arguable reflect tumour progenitor cell infiltration along superficial layers rather than co-existing dysplasia.

## 5 Conclusions

Our findings highlight differences in glial regenerative populations between dysplasia types of relevance to their divergent underlying aetiologies and potentially to their pathophysiology (Patel et al., 2019), warranting their further study.

## Declaration of Competing Interest

The authors report no declarations of interest.

## Figures and Tables

**Figure:**
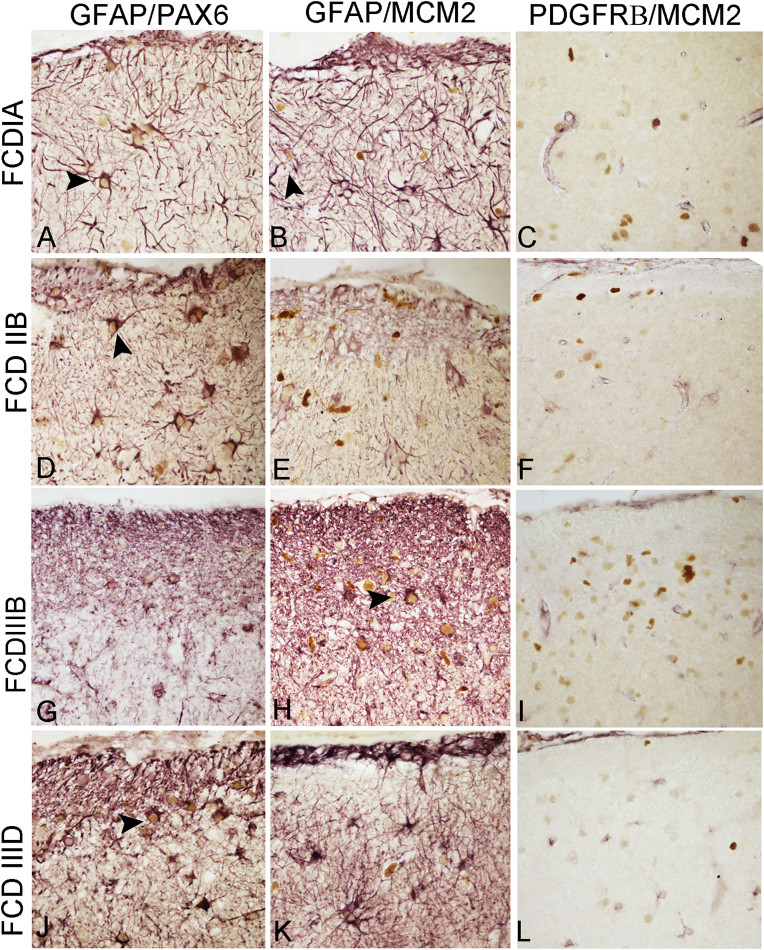

