# Supplementary material for: Glial regenerative cell types in the superficial cortex in cortical dysplasia subtypes
Source: Epilepsy Res. 2021 Jan;169:106529. doi: 10.1016/j.eplepsyres.2020.106529 (PMC7829594; doi:10.1016/j.eplepsyres.2020.106529)
Supplement: Supplementary file 1 [file mmc1.docx]

**Supplemental Figure.**

Representative images of superficial cortical layers of FCDIA (A-C), FCD2b (D-F), FCD3b (G-I), FCD3d (J-L) cases with double labelling for GFAP/PAX6 (A,D,G,J), GFAP/MCM2 (B,E,H,K) and PDGFRβ/MCM2 (C,F,I,L) showing single and double labelled (arrows) glial cells of similar morphology to that reported in FCD3a. All figures at objective magnification of x 40.
